# Molecular characterization of florfenicol and oxazolidinone resistance in *Enterococcus* isolates from animals in China

**DOI:** 10.3389/fmicb.2022.811692

**Published:** 2022-07-26

**Authors:** Pingping Li, Mengdi Gao, Chunlin Feng, Tielun Yan, Zhiqiong Sheng, Weina Shi, Shuang Liu, Lei Zhang, Anqi Li, Junwan Lu, Xi Lin, Kewei Li, Teng Xu, Qiyu Bao, Caixia Sun

**Affiliations:** ^1^Key Laboratory of Medical Genetics of Zhejiang Province, Key Laboratory of Laboratory Medicine, Ministry of Education China, School of Laboratory Medicine and Life Sciences, Wenzhou Medical University, Wenzhou, China; ^2^Nursing Department, The First Affiliated Hospital of Wenzhou Medical University, Wenzhou, China; ^3^Institute of Biomedical Informatics, School of Laboratory Medicine and Life Sciences, Wenzhou Medical University, Wenzhou, China; ^4^Department of Clinical Laboratory, Zhoukou Maternal and Child Health Hospital, Zhoukou, China; ^5^School of Nursing, Wenzhou Medical University, Wenzhou, China; ^6^Institute of Translational Medicine, Baotou Central Hospital, Baotou, China

**Keywords:** florfenicol, oxazolidinone, *fexA*, *fexB*, *optrA*, *poxtA*, *Enterococcus*, IS*1216*

## Abstract

Florfenicol is widely used for the treatment of bacterial infections in domestic animals. The aim of this study was to analyze the molecular mechanisms of florfenicol and oxazolidinone resistance in *Enterococcus* isolates from anal feces of domestic animals. The minimum inhibitory concentration (MIC) levels were determined by the agar dilution method. Polymerase chain reaction (PCR) was performed to analyze the distribution of the resistance genes. Whole-genome sequencing and comparative plasmid analysis was conducted to analyze the resistance gene environment. A total of 351 non-duplicated enteric strains were obtained. Among these isolates, 22 *Enterococcus* isolates, including 19 *Enterococcus. faecium* and 3 *Enterococcus. faecalis*, were further studied. 31 florfenicol resistance genes (13 *fexA*, 3 *fexB*, 12 *optrA,* and 3 *poxtA* genes) were identified in 15 of the 19 *E. faecium* isolates, and no florfenicol or oxazolidinone resistance genes were identified in 3 *E. faecalis* isolates. Whole-genome sequencing of *E. faecium* P47, which had all four florfenicol and oxazolidinone resistance genes and high MIC levels for both florfenicol (256 mg/L) and linezolid (8 mg/L), revealed that it contained a chromosome and 3 plasmids (pP47-27, pP47-61, and pP47-180). The four florfenicol and oxazolidinone resistance genes were all related to the insertion sequences IS*1216* and located on two smaller plasmids. The genes *fexB* and *poxtA* encoded in pP47-27, while *fexA* and *optrA* encoded in the conjugative plasmid pP47-61. Comparative analysis of homologous plasmids revealed that the sequences with high identities were plasmid sequences from various *Enterococcus* species except for the Tn*6349* sequence from a *Staphylococcus aureus* chromosome (MH746818.1). The current study revealed that florfenicol and oxazolidinone resistance genes (*fexA*, *fexB*, *poxtA,* and *optrA*) were widely distributed in *Enterococcus* isolates from animal in China. The mobile genetic elements, including the insertion sequences and conjugative plasmid, played an important role in the horizontal transfer of florfenicol and oxazolidinone resistance.

## Introduction

Enterococci are Gram-positive facultative anaerobes that typically colonize the gastrointestinal tracts of most animals and humans. However, along with staphylococci, enterococci are among the leading causes of nosocomial infections, such as bacteremia, peritonitis, endocarditis, urinary tract, wound, and device-related infections (Sava et al., 2010). Multidrug-resistant (MDR) enterococci are significant nosocomial pathogens that can develop resistance to many antimicrobials currently used in the clinic (Arias and Murray, 2012).

Florfenicol is a fluorinated derivative of chloramphenicol and has a broad-spectrum bacteriostatic activity for a wide range of microorganisms by binding to the peptidyl transferase center at the 50S ribosomal subunit of the 70S complex, which inhibits bacterial protein biosynthesis (Syriopoulou et al., 1981). In order to control the respiratory tract microbial infection of cattle and pigs, florfenicol has been licensed in Europe in 1995 and 2000, respectively (Schwarz and Chaslus-Dancla, 2001; Schwarz et al., 2004). China also approved its use in 1999 for the treatment of bacterial infection in pigs, cattle, chickens, and fish. Since then, florfenicol has been excessively used in veterinary medicine and as a feed additive, and the prevalence and abundance of florfenicol resistance genes has increased in bacteria from different sources (Zhao et al., 2016).

Oxazolidinones are antimicrobial agents that are effective in treating various Gram-positive bacterial infections as those caused by methicillin-resistant *Staphylococcus* (MRSA) and vancomycin-resistant enterococci (VRE; Fung et al., 2001). The most common mechanism contributing to linezolid resistance involves point mutations in domain V of the 23S rRNA gene, and it has been found that the most frequent mutation is on G2576T. The oxazolidinone-resistant phenotype is also related to spontaneous mutations in the ribosomal subunit proteins L3, L4, and L22 encoded by the *rplC*, *rplD*, and *rplV* genes, respectively (Long et al., 2006; Mendes et al., 2010). It was reported that the *cfr*, *cfr(B)*, *cfr(C)*, *cfr(D)*, *cfr(E)*, *optrA,* and *poxtA* genes are related to oxazolidinone resistance. The *cfr* gene is the first transferable oxazolidinone resistance gene, which encodes a rRNA methyltransferase (Schwarz et al., 2000; Deshpande et al., 2015; Wang et al., 2015; Tamang et al., 2017; Tang et al., 2017; Antonelli et al., 2018; Stojkovic et al., 2019). Homologs of *cfr* have been identified in nonpathogenic *Bacillales*, and three additional *cfr*-like genes that share less than 80% protein sequence identity to Cfr have been described in *Clostridium* and *Enterococcus*. These genes are known as *cfr(B)*, *cfr(C)*, and *cfr(D)*. A new determinant termed *cfr(E)* was recovered in *Clostridium difficile* strains across Latin America (Stojkovic et al., 2019). However, a novel ABC transporter gene *optrA* was first reported to be encoded in a plasmid from *Enterococcus faecalis* in 2015 in China (Wang et al., 2015). Since it was first described in 2015, *optrA* has been found in the human and animal enterococci, and it has environmental origins from Ireland, Italy, Korea, Malaysia, Denmark, and different regions of China, and it has been detected in pet food in Europe and in different sources from Tunisia, India, Czech Republic, and Sweden (Elghaieb et al., 2020; Bakthavatchalam et al., 2021; Fang et al., 2021; Freitas et al., 2021; Malisova et al., 2021). Furthermore, many *optrA* variants have been collected from enterococci and staphylococci (Cai et al., 2015; Brenciani et al., 2016; Cui et al., 2016; Li et al., 2016; Mendes et al., 2016; Cavaco et al., 2017; Lazaris et al., 2017; Lee et al., 2017). Recently, a novel oxazolidinone resistance gene named *poxtA*, encoding an ATP-binding cassette (ABC) protein, was identified in a MRSA that originated from the clinical (Antonelli et al., 2018).

Domestic animals are widely recognized as a source of antimicrobial resistance. Worldwide reports of *optrA*-mediated oxazolidinone resistance among bacterial isolates of animal origin have largely focused on animals from farms (Fan et al., 2016; Torres et al., 2018; Elghaieb et al., 2020). The wide distribution of oxazolidinone resistance *optrA* and *cfr* genes in domestic animals has been reported in rural areas of China (Sun et al., 2018). The occurrence of the linezolid resistance gene *optrA* in linezolid-resistant *E. faecalis* and *E. faecium* isolates from food animals and animal carcasses was first reported in Korea (Tamang et al., 2017).

In this study, we used multiple approaches, including molecular cloning, whole-genome sequencing, and comparative plasmid analysis, to investigate the molecular resistance mechanism against amphenicol and oxazolidinone from *Enterococcus* isolated from domestic animal samples in China.

## Materials and methods

### Bacterial isolates

Samples were collected from the anal feces of cattle, chickens, ducks, and pigs from five animal farms in Zhejiang (Hangzhou), Henan (Zhoukou), Shanxi (Linfen), Shandong (Liaocheng), and Sichuan (Bazhong) provinces in 2016, respectively. Only one farm was chosen in each sampling site. Then, the feces samples were streaked onto LB agar plates. A total of 351 non-duplicated enteric strains were obtained. Then, the 351 strains were cultured on LB agar plates supplemented with 8 mg/L florfenicol to screen the florfenicol-resistant strains. The strains were first identified by biochemical methods and then verified by homology comparisons of the 16S rRNA gene with the nucleotide sequence database by using BLASTN program^1^ (Altschul et al., 1990).

### Detection of resistance genes

The presence of resistance genes (*fexA*, *fexB*, *cfr,* and *optrA*) was investigated using PCR, as previously described (Kehrenberg and Schwarz, 2006; Liu et al., 2012; Wang et al., 2015). The primers (Table 1) were designed by using Primer 5.0 and synthesized by Shanghai Sunny Biotechnology Co., Ltd. (Shanghai, China), which included a pair of flanking restriction endonuclease adapters. PCR amplification was carried out under the following conditions: an initial 10 min denaturation at 94°C followed by 35 cycles of denaturation (94°C for 45 s), annealing (58°C for 45 s), and extension (72°C for 90 s), and a final extension step at 72°C for 10 min. The PCR products were further confirmed by Sanger sequencing with an ABI 3730 automated sequencer (Shanghai Sunny Biotechnology Co., Ltd., Shanghai, China). Both strands of the PCR products were sequenced with the forward and reverse primers (Table 1). The sequence data were compared to the NCBI nucleotide sequence database using the BLAST program (Altschul et al., 1990).

**Table 1:** Primer sequences and PCR product sizes of the florfenicol and oxazolidinones resistance genes.

Resistance gene	Accession number	Sequence (5′ → 3′)	Restriction endonuclease	Amplicon size (bp)	Annealing temperature (°C)
*fexA*	NG_047857	CGGGATCCATGAAAAAGGATAGTAAATC	*BamH* I	1,428	58
		GCTCTAGATTACCCACGCTTTTTTAAACC	*Xba* I		
*fexB*	JN201336.1	CGGAATTCATGAATCATCAAAATGAAAAAAATA	*EcoR* I	1,410	58
		CCAAGCTTTTATTGCTTTAAATCCCTACTACTT	*Hind* III		
*optrA*	NG_048023	GGGGTACCTTGTCCAAAGCCACCTTTGCAATTGCTAGT	*Kpn* I	1,968	58
		GCGTCGACTTACATAACTTCCAATTCTCTCATCAACTG	*Sal* I		
*cfr*	AJ249217.1	CGGAATTCATGAATTTTAATAATAAAACAAAGTATGG	*EcoR* I	1,050	58
		CCAAGCTTCTATTGGCTATTTTGATAATTACCATATA	*Hind* III		
*optrA-promotor*	NG_048023	GCTCTAGATTTCTCACCCAGATATGCCCGGGATCCCGGCAAACTCAAAAGGTC	*Xba* I	2,582	63

Underlined sequences are restriction endonuclease sites.

### Pulsed field gel electrophoresis

Pulsed field gel electrophoresis (PFGE) was carried out on *Enterococcus* isolates according to the method described previously with minor modifications (Liu et al., 2013). Enterococcal cells were treated with lysozyme at a final concentration of 10 mg/ml for 1 h at 37°C and then incubated with the restriction enzyme *Sma*I (Takara, Japan) for 4 h with shaking. Briefly, genomic DNA fragments were resolved on a 1% agarose (SeaKem Gold Agarose, Lonza) gel at 14°C, and electrophoresis was conducted using a CHEF-Mapper XA PFGE system (Bio-Rad, United States) at a pulse time gradient of 3.5–23.5 s and a total running time of 18 h within a molecular weight of 10–300 kb. The DNA band patterns were visualized by a Gel Doc XR gel imaging system (Bio-Rad, United States) and further analyzed by QualityOne software (Bio-Rad Laboratories, United States). Phylogenetic trees then allow the comparison of genetic relatedness and clonal assignment (Neoh et al., 2019). A similarity coefficient of 80% was selected to define the pulsed-field type (PFT) clusters (McDougal et al., 2003).

### Whole-genome sequencing and multi-locus sequence typing

Due to *E. faecium* P47 showing high resistance to florfenicol and linezolid and carrying the most resistance genes, it was selected for whole-genome sequencing. The genomic DNA of *E. faecium* P47 was extracted by an AxyPrep Bacterial Genomic DNA Miniprep kit (Axygen Scientific, Union City, CA, United States) according to the manufacturer’s instructions. A 20-kb library was generated using the SMRTbell Template Prep Kit (Pacific Biosciences, CA, United States) according to the PacBio standard protocol and was sequenced on a PacBio RS II instrument (Pacific Biosciences). In addition, a paired-end library with 300-bp insert sizes was constructed and sequenced from both ends using the HiSeq 2,500 Illumina sequencing platform (Illumina, CA, United States). The PacBio long reads were initially assembled using Canu software (Koren et al., 2017). The Illumina sequencing reads were then mapped onto the assembled contigs to correct the primary assembly by using Bwa and the Genome Analysis Toolkit (McKenna et al., 2010). The potential ORFs were predicted using Glimmer software and annotated against a non-redundant protein database using the BLASTX program (Altschul et al., 1990).

Genotyping by the multi-locus sequence typing (MLST) method for *E. faecium* P47 was determined using 7 housekeeping genes (*atpA*, *ddl*, *gdh*, *purK*, *gyd*, *pstS,* and *adk*). Alleles and sequence types (STs) were assigned using the MLST database^2^ (Homan et al., 2002).

### Plasmid transfer and cloning experiments

The transferability of linezolid and florfenicol resistance was assessed by filter mating using *E. faecium* P47 as a donor and *E. faecalis* JH2-2 as a recipient, following the method described previously (Huys et al., 2004). The transconjugants were selected on brain heart infusion (BHI) plates containing 16 mg/L florfenicol, 25 mg/L rifampicin, and 50 mg/L fusidic acid. The size of the transferred plasmid was estimated by S1 nuclease-PFGE as previously reported with some modifications (Liu et al., 2013). Whole-cell DNA was incubated with 32 U S1-nuclease (TaKaRa, Japan) for 25 min at 30°C. The resulting restriction fragments were separated using a CHEF-DRIII system (Bio-Rad) with a clamped homogeneous electric field of 6 V/cm, using a 120° switch angle for 17 h at 14°C, with the pulse time linearly ramped from 3.5 to 23.5 s. PCR amplifications of the *fexA*, *fexB, poxtA* and *optrA* genes were performed to confirm its presence in the transconjugants.

To confirm the function of the *optrA* gene in *E. faecium* P47, open reading frames with upstream promoter regions were amplified using the *optrA* promoter primers (Table 1). The purified PCR products were digested and ligated into the shuttle vector pAM401, and further transformed into *E. faecalis* JH2-2 by electrotransformation and grown on BHI plates supplemented with 16 mg/L chloramphenicol. The recombinant plasmids were verified by *Xba*I and *BamH*I (TaKaRa, Japan) digestion and then sequencing.

### Antimicrobial susceptibility testing

The MICs for 22 *Enterococcus* isolates, transconjugants, and transformants were determined by the agar dilution method following the guidelines of the Clinical and Laboratory Standards Institute (CLSI, 2021: M100).^3^ A total of 16 antimicrobial agents, including chloramphenicol, florfenicol, linezolid, ampicillin, gentamicin, erythromycin, tetracycline, vancomycin, fosfomycin, rifampicin, fusidic acid, spectinomycin, norfloxacin, levofloxacin, clarithromycin, and tigecycline, were used. *Enterococcus faecalis* ATCC 29212 was used as a quality control strain.

### Comparative plasmid analysis

The sequences used for the comparative plasmid analysis of pP47-27 or pP47-61 were retrieved from the NCBI nucleotide database using the complete nucleotide sequence of pP47-27 or pP47-61 as a query based on a cutoff value of 40% coverage and 97% identity for pP47-61 and 90% coverage and 99% identity for pP47-27. Plasmid incompatibility groups were predicted by PlasmidFinder^4^ (Carattoli et al., 2014). Nucleotide sequence comparisons were carried out using BLASTN. Gview^5^ was used to construct basic genomic features that were then employed in comparative plasmid analysis (Petkau et al., 2010). For the comparative plasmid analysis of the *optrA*-encoding fragment, sequences containing the *optrA* gene were also obtained from the NCBI nucleotide database using “*optrA* gene” as a keyword. The resulting sequences were filtered, and only sequences that contained a complete *optrA* gene and harbored at last one complete or truncated IS*1216* gene were retained. The plasmid pE349 which was first reported to carry *optrA* gene was also retained. Multiple sequence alignments were performed by MAFFT using the 16 kb *optrA* gene-related fragment (approximately 8 kb upstream and downstream of the *optrA* gene) of pP47-61 as a reference (Katoh and Standley, 2013). The 16 kb fragment was flanked by two IS*1216* insertion sequences. The sequence with the highest sequence similarity to the other sequences in each cluster was chosen as the candidate for ortholog analysis. Orthologous groups of genes from the candidate sequences were identified using BLASTP and InParanoid (Altschul et al., 1990; Remm et al., 2001). The sequence retrieval, statistical analysis, and other bioinformatic tools used in this study were applied with Python and Biopython scripts (Cock et al., 2009).

### Nucleotide sequence accession numbers

The genome sequence of P47 has been deposited in GenBank with the following accession numbers CP091100 (P47 chromosome), CP091101 (pP47-27), CP091102 (pP47-61), and CP091103 (pP47-180).

## Results

### Antibiotic resistance and resistance gene detection of the isolates

We collected a total of 351 non-duplicated enteric bacterial strains in five farms from five provinces of China, including 237 *Escherichia coli*, 45 *Bacillus*, 38 *Staphylococcus*, 22 *Enterococcus* (3 *Enterococcus faecalis* and 19 *Enterococcus faecium*), 4 *Klebsiella pneumoniae*, 4 *Shigella*, 4 *Planctomycetes*, 3 *Proteus,* and 4 unclassified strains. The source and regional distribution of 351 strains are shown in Supplementary Table S1. We have investigated the resistance profile of animal-derived Gram-negative strains in previous studies (Wu et al., 2019; Li et al., 2020; Liu et al., 2020; Zhu et al., 2020). In this study, we mainly focused on the resistance characteristics of *Enterococcus* in the animals. Therefore, 22 *Enterococcus* strains, including 19 *E. faecium* and 3 *E. faecalis*, were subjected to further antimicrobial resistance investigation. Among 22 *Enterococcus* isolates, most (81.8%, 18/22) were isolated from chickens, and the remaining 4 strains were pig-origin. More than 68.2% (15/22) of the strains showed resistance to 5 antibiotics, including chloramphenicol (77.3%, 17/22), florfenicol (77.3%, 17/22), erythromycin (68.2%, 15/22), tetracycline (81.8%, 18/22), and clarithromycin (90.9%, 20/22), while only 2 strains were resistant to linezolid (9.1%, 2/22), as shown in Table 2.

**Table 2:** Characterization of the susceptibility of 22 *Enterococcus* isolates to 16 antibiotics.

Antibiotics	*E. faecium* (*N* = 19)			*E. faecalis* (*N* = 3)			Total (*N* = 22)		
	*S*	*I*	*R*	*S*	*I*	*R*	*S*	*I*	*R*
CHL	2 (10.5%)	0	17 (89.5%)	3 (100%)	0	0	5 (22.7%)	0	17 (77.3%)
FFC	2 (10.5%)	0	17 (89.5%)	3 (100%)	0	0	5 (22.7%)	0	17 (77.3%)
LZD	6 (31.6%)	11 (57.9%)	2 (10.5%)	3 (100%)	0	0	9 (40.9%)	11 (50.0%)	2 (9.1%)
AMP	19 (100%)	0	0	3 (100%)	0	0	22 (100%)	0	0
GEN	16 (84.2%)	0	3 (15.8%)	3 (100%)	0	0	19 (86.4%)	0	3 (13.6%)
SPT	9 (47.4%)	0	10 (52.6%)	3 (100%)	0	0	12 (54.5%)	0	10 (45.5%)
NOR	3 (15.8%)	6 (31.6%)	10 (52.6%)	3 (100%)	0	0	6 (27.3%)	6 (27.3%)	10 (45.5%)
LVX	9 (47.4%)	0	10 (52.6%)	3 (100%)	0	0	12 (54.5%)	0	10 (45.5%)
ERY	0	4 (21.1%)	15 (78.9%)	0	3 (100%)	0	0	7 (31.8%)	15 (68.2%)
CLA	0	1 (5.3%)	18 (94.7%)	0	1 (33.3%)	2 (66.7%)	0	2 (9.1%)	20 (90.9%)
TET	1 (5.3%)	0	18 (94.7%)	3 (100%)	0	0	4 (18.2%)	0	18 (81.8%)
TGC	19 (100%)	0	0	3 (100%)	0	0	22 (100%)	0	0
VAN	16 (84.2%)	1 (5.3%)	2 (10.5%)	3 (100%)	0	0	19 (86.4%)	1 (4.5%)	2 (9.1%)
FOS	17 (89.5%)	2 (10.5%)	0	2 (66.7%)	1 (33.3%)	0	19 (86.4%)	3 (13.6%)	0
RIF	5 (26.3%)	0	14 (73.7%)	0	0	3 (100%)	5 (22.7%)	0	17 (77.3%)
FD	16 (84.2%)	0	3 (15.8%)	0	0	3 (100%)	16 (72.7%)	0	6 (27.3%)

CHI, Chloramphenicol; FFC, Florfenicol; LZD, linezolid; AMP, Ampicillin; GEN, Gentamicin; SPT, Spectinomycin; NOR, Norfloxacin; LVX, Levofloxacin; ERY, Erythromycin; CLA, Clarithromycin; TET, Tetracycline; TGC, Tigecycline; VAN, Vancomycin; FOS, Fosfomycin; RIF, Rifampicin; FD, Fusidic acid

A total of 31 florfenicol and oxazolidinone resistance genes (including 13 *fexA*, 3 *fexB*, 12 *optrA,* and 3 *poxtA* genes) were identified in 15 of the 19 *E. faecium* isolates, and no florfenicol or oxazolidinone resistance genes were identified in any of the *E. faecalis* isolates. In the resistance gene-positive isolates, more than two-thirds (72.3%, 11/15) carried two genes, most of which (10/11, 90.1%) carried *fexA* and *optrA,* and one strain (*E. faecium* FH67) harbored *fexB* and *poxtA* simultaneously. Among the other four isolates, two strains (*E. faecium* FH56 and *E. faecium* FH58) carried *fexA* each, one strain (*E. faecium* FH59) carried three genes (*fexB, optrA,* and *poxtA*), and one strain (*E. faecium* P47) carried four genes (*fexA*, *fexB*, *optrA,* and *poxtA*). All the florfenicol-resistant isolates (77.3%, 17/22), which exhibited MIC levels ranging from 64–2048 mg/L, were *E. faecium*, while 5 isolates including 2 *E. faecium* and 3 *E. faecalis* isolates were susceptible to florfenicol and exhibited MIC levels (≤ 4 mg/L). None of the known florfenicol resistance genes were identified in the 5 florfenicol-susceptible isolates. However, two florfenicol-resistant isolates which did not harbor any known florfenicol resistance gene had a MIC of 256 mg/L (H38) and 64 mg/L (FH72), respectively (Supplementary Table S2).

The florfenicol resistance gene-positive strains also showed higher MIC levels to linezolid than those strains which did not contain florfenicol resistance genes. A total of 86.7% (13/15) of florfenicol resistance gene-positive strains (excluding FH75 and H32 which had MIC levels of 2 mg/l with linezolid) exhibited MIC levels of ≥4 mg/L against linezolid; however, in the florfenicol resistant gene-negative strains, none of the strains exhibited this MIC level against linezolid.

### Molecular typing by pulsed field gel electrophoresis

**Figure 1:**
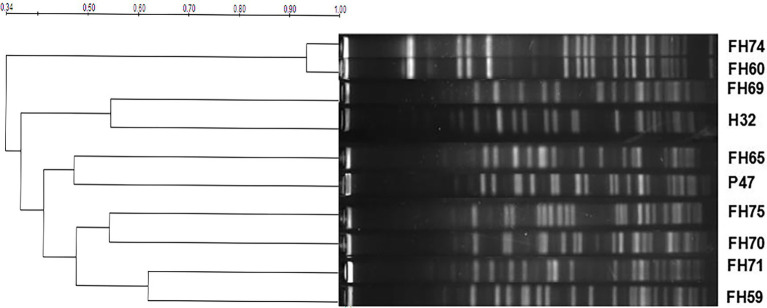
PFGE patterns of 10 *optrA*-positive *E. faecium* isolates.

Pulsed field gel electrophoresis (PFGE) typing for 10 *optrA*-positive *E. faecium* strains was analyzed. The band sizes were mainly between 20–700 kb, and the number of bands was approximately 15–22. Based on a similarity coefficient of 0.80, the 10 *E. faecium* isolates showed 9 pulsed-field type (PFT) clusters (Figure 1). There were two isolates (FH74 and FH60) harboring the same florfenicol resistance genes (*optrA* and *fexA*) from the same chicken farm in Hangzhou, China, sharing over 90% similarity. They could be considered the same PFT, which indicated that there may be a clone spread of the strains harboring the *optrA* and *fexA* genes in the farm.

### General features of the *Enterococcus faecium* P47 genome

**Figure 2:**
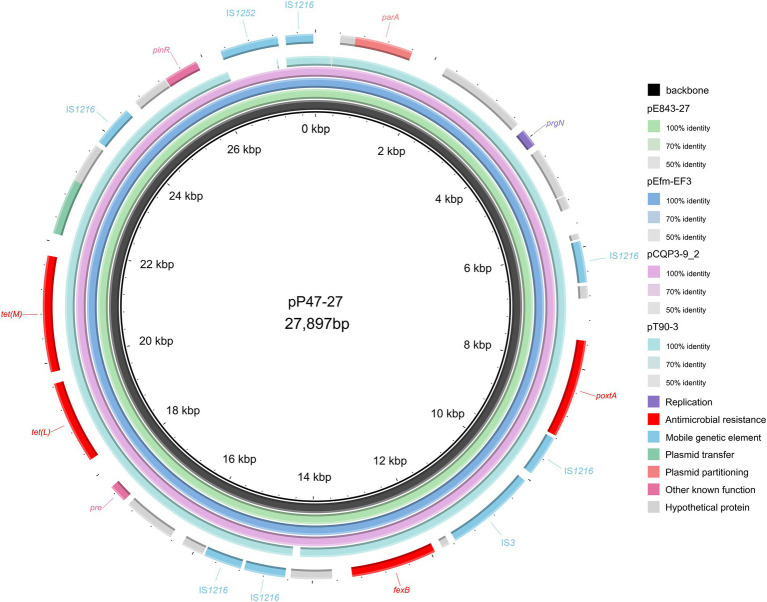
Comparative plasmid analysis of pP47-27 and other similar plasmids. The inside to the outside is as follows: circle 1, the backbone of pP47-27; circle 2, pE843-27 (the plasmid of *Enterococcus lactis* E843 isolated from swine, CP082268.1); circle 3, pEfm-EF3 (the plasmid of *Enterococcus faecium* EF3 isolated from marine sediment, MT683615.1); circle 4, pCQP3-9_2 (the plasmid of *Enterococcus hirae* pCQP3-9 isolated from fecal sample, CP037957.1); circle 5, pT90-3 (the plasmid of *Enterococcus faecalis* strain T90-3 isolated from swine, CP069131.1); and circle 6, the genes encoded on pP47-27.

**Figure 3:**
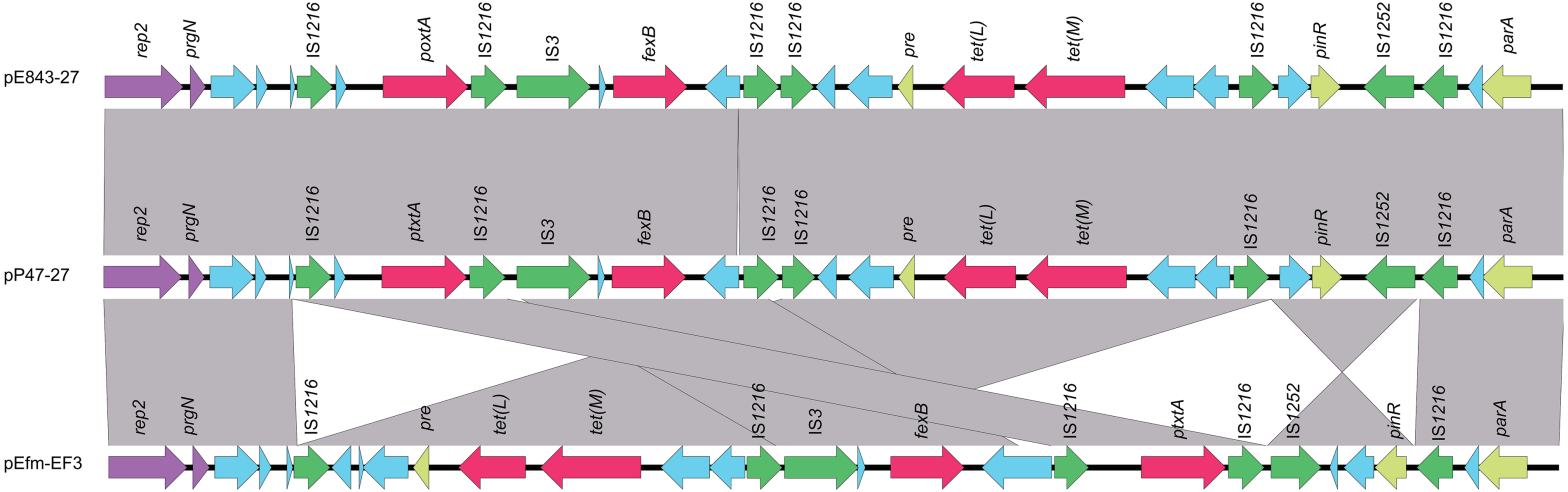
Genetic environments of the *poxtA* and *fexB* genes encoded in different plasmids. The ORFs are shown as arrows, and the arrowheads indicate the direction of transcription. The same color represents the same elements and genes, with antimicrobial resistance genes in red, mobile genetic elements in green, replications in purple, and other genes in blue. Gray-shaded areas represent regions with >95% nucleotide sequence identities. The sequences and their origins are *Enterococcus lactis* E843 (the plasmid of *Enterococcus lactis* E843 isolated from swine, CP082268.1) and *Enterococcus faecium* EF3 (the plasmid of *Enterococcus faecium* EF3 isolated from marine sediment, MT683615.1).

**Figure 4:**
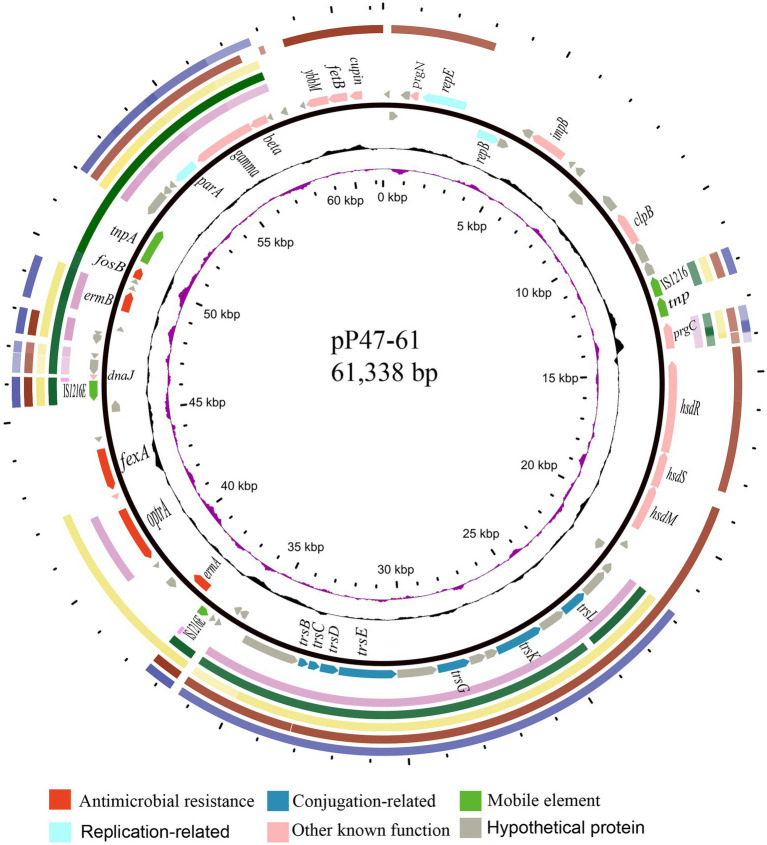
Comparative plasmid analysis of pP47-61 and other similar sequences. Circles from outside to inside indicate (1) the regions of the four plasmids (all from *Enterococcus,* including pCR1B, CP030934.1; pF120805, KY579372.1; pKUB3007-3, AP018546.1; and pE35048-oc, MF580438.1) and a transposon sequence of the *Staphylococcus aureus* AOUC-0915 chromosome (Tn*6349,* MH746818.1), which share high homology with pP47-61. (2) Predicted coding sequences encoded on the forward and reverse strands of pP47-61. (3) G + C content (with an average of 50%, in which a G + C content of more than 50% is shown toward the outside and less is shown inward) and G + C skew (with a positive GC skew toward the outside and a negative GC skew toward the inside) of pP47-61. (4) The innermost circle shows the position in kbp.

**Figure 5:**
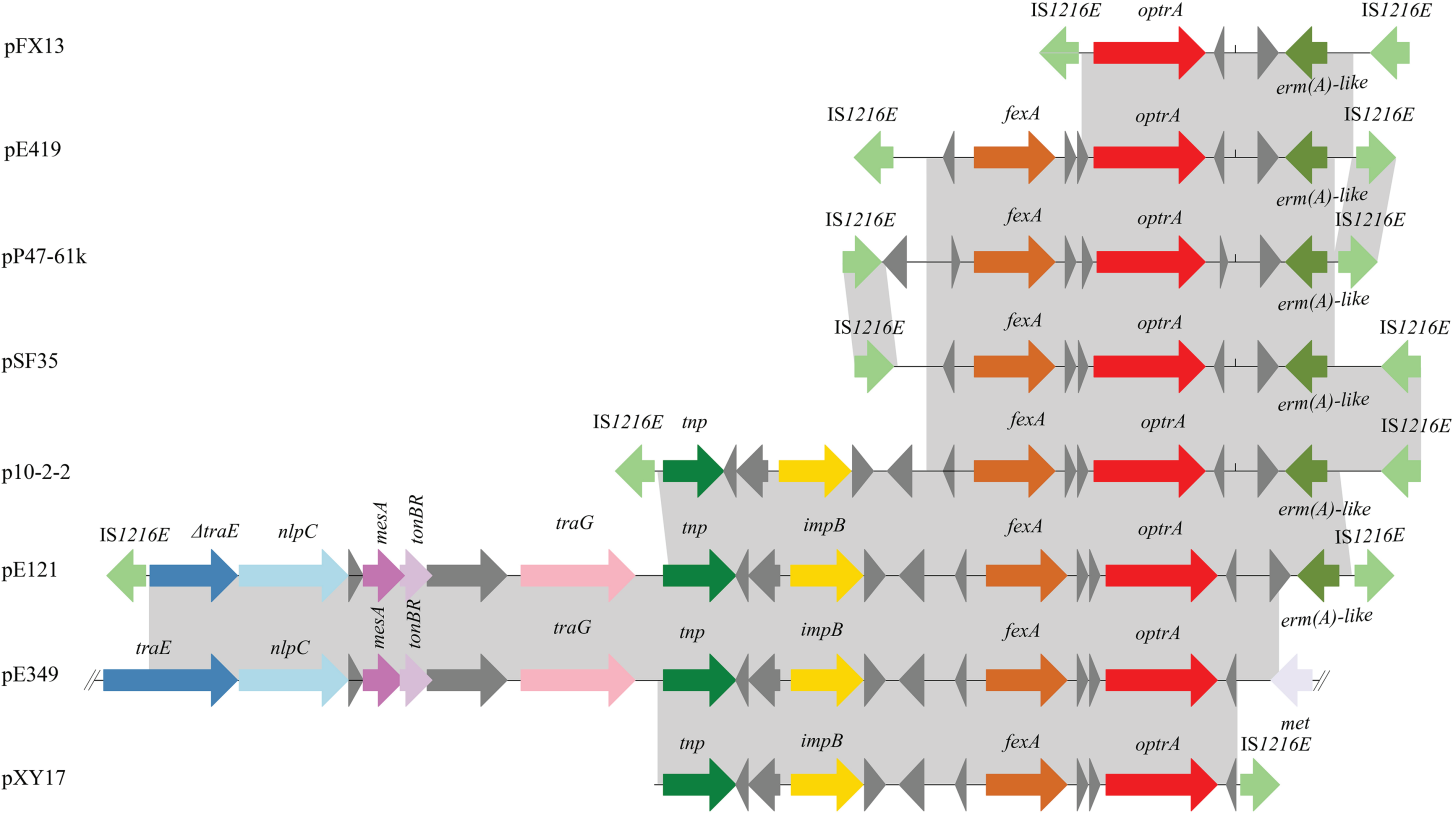
Schematic representation of the genetic environments surrounding the *optrA* gene in different sequences. The ORFs are shown as arrows, with the arrowhead indicating the direction of transcription. The same color represents the same elements and genes. Regions are drawn to scale. The sequences were retrieved from GenBank with accession numbers KT862775.1 (p10-2-2), T862776.1 (pE121), KP399637.1 (pE394), KT862777.1 (pE419), KT862778.1 (pFX13), KT862779.1 (pSF35), and KT862780.1 (pXY17).

To elucidate the molecular resistance mechanism of the *Enterococcus* strains to antimicrobials, especially florfenicol and oxazolidinones, the whole-genome sequence of *E. faecium* P47 was chosen for whole-genome sequencing (WGS) analysis. *E. faecium* P47 co-carries four florfenicol and oxazolidinone resistance genes, *fexA, fexB, optrA,* and *poxtA,* and has a wide resistance spectrum and high MIC values to the antibiotics tested (especially linezolid with an MIC level of 8 mg/L). The general features of the P47 genome are shown in Table 3. The complete *E. faecium* P47 genome consisted of one chromosome and three circular plasmids (pP47-27, pP47-61, and pP47-180). The chromosome had an average G + C content of 38.12% and was 2,560,635 bp in length, encoding 2,424 ORFs. Of the three plasmids, pP47-27 was 27,897 bp in length, encoding 29 ORFs; pP47-61 was 61,338 bp in length, encoding 64 ORFs; and pP47-180 was 180,523 bp in length, encoding 204 ORFs. Functional annotation revealed that the whole genome encoded 14 resistance genes with 2 (*aac(6′)-Ii* and *dfrG*) in the chromosome and 12 in the plasmids, including 4 (*fexB*, *poxtA*, *tetL* and *tetM*) encoded on the plasmid pP47-27 (Figures 2, 3), 5 (*ermB*, *fosB*, *ermA*, *optrA* and *fexA*) on the plasmid pP47-61 (Figure 4) and 3 [*aac(6′)-aph(2″)*, *ant(6)-Ia* and *ermB*] on the plasmid pP47-180 (Supplementary Table S2). MLST analysis revealed that *E. faecium* P47 contained the *adk*-1, *atpA*-4, *ddi*-5, *gdh*-1, *gyd*-1, *pstS*-1, and *purK*-3 alleles and belonged to the sequence type ST29.

**Table 3:** General features of the genome of *E. faecium* P47.

	Chromosome	pP47-27	pP47-61	pP47-180
Size (bp)	2,560,635	27,897	61,338	180,523
GC content (%)	38.12	35.44	34.24	35.18
Total opening reading frames	2,424	29	64	204
Known proteins	1,924	21	38	140
Hypothetical proteins	500	8	26	64
Protein coding sequence (%)	84.76	76.90	80.03	80.37
Average ORF length (bp)	895	740	767	711
rRNA operons	2*(16 s-23 s-5 s)			
tRNA	68			

### Transferability of the resistance plasmid and resistance activity of the transconjugant

Through conjugation experiments, a transconjugant carrying transferable pP47-61 was obtained and further determined by S1 nuclease-PFGE. The MIC results revealed that the transconjugant (pP47-61/JH2-2) exhibited at least a 4-fold increase in MIC levels for florfenicol, chloramphenicol, and linezolid and a > 512-fold increase in MIC levels for macrolides (erythromycin and clarithromycin) compared to that of the recipient strain JH2-2, which was in accordance with the resistance genes (*fexA*, *optrA*, *ermA,* and *ermB*) encoded on the plasmid (Supplementary Table S2).

The WGS sequencing results demonstrated that the deduced OptrA protein from the plasmid pP47-61 included three amino acid substitutions at position 3 (K3E), 176(Y176D), and 481(T481P; EDP variant) compared with the first reported *optrA* gene in *E. faecalis* E349 of human origin (Wang et al., 2015). To identify the resistance activity of the *optrA* gene (EDP variant) of *E. faecium* P47, the ORF with its promoter region was cloned. The MIC levels of the recombinant strain expressing the cloned resistance gene against 16 antimicrobial agents are shown in Supplementary Table S2. The recombinant strain harboring the cloned *optrA* (*optrA-*pAM401/JH2-2) exhibited 8-and 2-fold increases in MIC levels with florfenicol and linezolid compared to the control strain JH2-2/pAM401, respectively, while no MIC changes were found for the other antibiotics tested compared with the control strain JH2-2/pAM401 (*E. faecalis* JH2-2 harboring the vector pAM401).

### Comparative plasmid analysis and the genetic environment of the resistance genes on the plasmids

Four plasmids and a transposon sharing the highest nucleotide sequence similarity (≥ 40% coverage and ≥ 97% identity) with pP47-61 were retrieved from the NCBI nucleotide database. The plasmids belonged to the Inc18 incompatibility group, including pCR1B of *E. gilvus* CR1 (CP030934.1, 80 kb) isolated from Japan, pF120805 of *E. faecium* F120805 (KY579372.1, 72 kb) from Ireland, pKUB3007-3 of *E. faecalis* KUB3007 (AP018546.1, 59 kb) from Japan, pE35048-oc of *E. faecium* E35048 (MF580438.1, 41 kb) from Italy, and Tn*6349* from *Staphylococcus aureus* AOUC-0915 chromosome (MH746818.1, 48 kb) from Italy (Morroni et al., 2018; D’Andrea et al., 2019). The plasmids displayed highly global genomic synteny and shared a conserved backbone sequence of a typical Inc18 plasmid, which included two homologous regions (regions A and B, Figure 4; Supplementary Table S3). Region A, approximately 10 kb in size (from 23 to 37 kb), contained a cluster of genes related to conjugation (*trsBCDEGKL*) and a series of hypothetical proteins. Region B, approximately 10 kb in size (from 45 to 57 kb), encoded genes of *dnaJ* (a molecular chaperone), *ermB*, and *parA* (for partition), and this region was interrupted by the insertion of *tnpA* (transposase) and *fosB* (fosfomycin resistance enzyme) into plasmids pP47-61 and pKUB3007-3. The segment (from 37 to 45 kb) of an approximately 8.0 kb sequence encoding a complex transposon with a gene array of IS*1216E*-hp-*fexA-hp*-*optrA*-hp-hp-*ermA*-IS*1216E* was unique in pP47-61, although part of the sequence was found in the plasmids pF120805 and pE35048-oc. Comparative genomic analysis of the complex transposon encoding resistance genes (*fexA, optrA,* and *ermA*) of pP47-61 with similar sequences from the NCBI nucleotide database revealed that the core gene array of the complex transposon (hp-*fexA-hp*-*optrA*-hp-hp-*ermA*) was virtually identical to the sequences in the plasmids pE419 (*E. faecalis*E419), pSF35 (*E. faecalis*SF35), p10-2-2 (*E. faecalis* 10-2-2), and pE121 (*E. faecalis* E121), while in the other plasmid sequences (pFX13 of *E. faecalis*, pE121 of *E. faecalis* and pE349 of *E. faecalis*), they shared similar sequence segments (Figure 5; Supplementary Table S3).

When searching for sequences similar to plasmid pP47-27, we retrieved 25 sequences with identities >99.0% and coverage >90.0% from the NCBI nucleotide database (Supplementary Table S3). They were all plasmid sequences from the genus *Enterococcus*, including species of *Enterococcus faecium* (*n* = 17), *Enterococcus faecalis* (*n* = 5), *Enterococcus hirae* (*n* = 2), and *Enterococcus lactis* (*n* = 1). Of the 25 sequences, only two (pE843-27 from *E. lactis* and pEfm-EF3 of *E. faecium*) had a similar length of approximately 27 kb, while all the other plasmids were longer than 32 kb. Comparative genomic analysis revealed that all sequences encoded the florfenicol resistance genes *fexB* and *poxtA*, although mostly with different gene contexts or gene orders (Figures 2, 3; Supplementary Table S3).

## Discussion

In this work, 77.3% (17/22) and 9.1% (2/22) of the *Enterococcus* isolates tested were resistant to florfenicol and linezolid, respectively. This result is similar to the results that showed resistance rates of 78.3% (for florfenicol) and 11.3% (for linezolid) against 106 enterococcal isolates from pigeon and duck feces in Egypt (Osman et al., 2019). Most of the florfenicol-resistant isolates also showed resistance to the other four antimicrobials, chloramphenicol, erythromycin, clarithromycin, and tetracycline. These antimicrobials are commonly consumed in the poultry industry as animal growth additives or therapeutic drugs (Cheng et al., 2014). It was significant to discover that two isolates (FH69 and P47) showed resistances to linezolid (≥8 mg/L). Linezolid is a very important antimicrobial used in the treatment of human enterococcal infections and is constantly being monitored by surveillance programs such as the global Zyvox Annual Appraisal of Potency and Spectrum (ZAAPS; Mendes et al., 2016). The lower level of linezolid resistance among enterococci could be due to resistance genes (*optrA* and *poxtA*) and commonly found spontaneous mutations, such as the G2576T mutation in 23S rRNA (Biedenbach et al., 2010; Patel et al., 2013). Enterococci contain 4–6 copies of the 23S rRNA gene, and therefore, mutations in multiple copies are necessary to confer resistance (Biedenbach et al., 2010).

None of the enterococcal isolates in this work carried a *cfr* gene, while more than half (54.5%, 12/22) contained *optrA* genes. The *optrA-*positive rate was much higher than that reported by Kang et al. (2019), who found that only 3.7% (6/158) of enterococcal strains of swine origin harbored *optrA*. All of the *optrA*-positive isolates also co-carried the amphenicol exporter gene *fexA*. The presence of both *optrA* and *fexA* in the same isolate has been described in *Enterococcus* from humans and animals from China (Cai et al., 2015; Wang et al., 2015). To the best of our knowledge, this is the first report of one *E. faecium* isolate, P47, carrying four different amphenicol and oxazolidinone resistance genes (*fexA*, *optrA, fexB,* and *poxtA*). The most recently described linezolid resistance gene, *poxtA*, in *S. aureus* encodes a protein that is 32% identical to OptrA. Expression of *poxtA* in *E. faecalis* was able to decrease susceptibility to phenicols and oxazolidinones (Antonelli et al., 2018). As reported previously, *Enterococcus* isolates in the present study simultaneously carried several resistance genes that conferred the host bacterium resistance phenotypes, such as the *tet*(M) and *tet*(L) genes for tetracycline resistance and the *erm*(A) and *erm*(B) genes for macrolide-lincosamide-streptogramin B resistance (Almeida et al., 2020). Oxazolidinones are not approved for veterinary use in China, whereas tetracyclines, macrolides, and florfenicol are consistently being used in swine and poultry production in China (Kang et al., 2019). As mentioned above, florfenicol has been widely used for the treatment of gastrointestinal tract and respiratory infections in food-producing animals. The use of florfenicol has caused selective pressure and has likely been utilized for resistance carriage by some common constituent of the swine intestinal flora (Almeida et al., 2020). Therefore, the prevalence of *optrA* and *poxtA* in *Enterococcus* originating from household animals in the current study may be explained by co-selection. In particular, the regions encoding *optrA* and *poxtA* harbor multiple antimicrobial resistance genes. Selection for any of these resistance genes could also result in co-selection for the genes (Sun et al., 2018).

The resistance genes encoded in both pP47-27 [*poxtA*, *fexB*, *tet*(L), and *tet*(M)] and pP47-61 (*ermA, optrA, fexA, ermB,* and *fosB*) were all related to mobile genetic elements (MGEs). Comparative genomic analysis demonstrated that the similar genetic context of the resistance genes has been found in many plasmids (or chromosomes) of different bacterial species with animal and human origins (He et al., 2016; Kang et al., 2019), which indicated that mobile genetic elements participated in spreading the resistance genes, including amphenicol and oxazolidinone resistance genes, among the DNA molecules of different bacteria.

## Conclusion

In conclusion, the results of this study demonstrated that the occurrence of oxazolidinone and amphenicol resistance is still frequent among *Enterococcus* isolates from animals. Mobile genetic elements, such as conjugative plasmids, insertion sequences, and transposons, may facilitate the horizontal transmission of oxazolidinone and amphenicol resistance genes. The presence of other resistance genes, including *tet*(L), *tet*(M), *ermA,* and *ermB* encoded on the plasmids, may lead to their co-selection. Therefore, the use of amphenicol antibiotics in food-producing animals should be closely monitored. Livestock husbandry might be recognized as a source of transferable oxazolidinone and amphenicol resistance determinants that could be spread to humans through the food chain.

## Data availability statement

The datasets presented in this study can be found in online repositories. The names of the repository/repositories and accession number(s) can be found at: GenBank, accession numbers CP091100-CP091103.

## Ethics statement

The animal study was reviewed and approved by Animal Welfare and Ethics Committee of Wenzhou Medical University, Zhejiang Province, China (Animal protocol number: wydw2021-0323).

## Author contributions

PL, MG, WS, TY, SL, and XL collected the strains and performed the experiments. CF, LZ, JL, and TX analyzed the experimental results. CF and AL performed the bioinformatics analysis. PL, TX, KL, JL, and QB co-led the writing of the manuscript. QB and CS designed the work. All authors contributed to the article and approved the submitted version.

## Funding

This study was supported by the Science and Technology Project of Wenzhou City, China (N20210001), Zhejiang Provincial Natural Science Foundation of China (LY19C060002 and LQ17H190001), and the National Natural Science Foundation of China (81960381).

## Conflict of interest

The authors declare that the research was conducted in the absence of any commercial or financial relationships that could be construed as a potential conflict of interest.

## Publisher’s note

All claims expressed in this article are solely those of the authors and do not necessarily represent those of their affiliated organizations, or those of the publisher, the editors and the reviewers. Any product that may be evaluated in this article, or claim that may be made by its manufacturer, is not guaranteed or endorsed by the publisher.

## Footnotes

^1^ https://blast.ncbi.nlm.nih.gov/blast.cgi

^2^ https://pubmlst.org/efaecium/

^3^ https://clsi.org/standards/

^4^ https://cge.cbs.dtu.dk//services/

^5^ https://stothard.afns.ualberta.ca/cgview_server/
