# Supplementary material for: Molecular characterization of florfenicol and oxazolidinone resistance in Enterococcus isolates from animals in China
Source: Front Microbiol. 2022 Jul 26;13:811692. doi: 10.3389/fmicb.2022.811692 (PMC9360786; doi:10.3389/fmicb.2022.811692)
Supplement: Supplementary file 1 [file Table_4.DOC]

**TABLE S1 |**  The region sources and sample sources of 351 strains

| Source  Region | pig | chicken | cattle | duck | Total |
| --- | --- | --- | --- | --- | --- |
| Zhejiang | 81 | 56 | 20 | 12 | 169 |
| Sichuan | 12 | 13 | 0 | 18 | 43 |
| Henan | 40 | 29 | 6 | 0 | 75 |
| Shandong | 30 | 11 | 0 | 6 | 47 |
| Shanxi | 17 | 0 | 0 | 0 | 17 |
| Total | 180 | 109 | 29 | 33 | 351 |
